# Distressed or De-stressed? Crafting “Mental Health” in University Peer Education and Health Promotion

**DOI:** 10.1007/s11013-026-10001-8

**Published:** 2026-07-11

**Authors:** Gracie Wilson

**Affiliations:** Department of Comparative Human Development, University of Chicago, Chicago, United States

**Keywords:** University mental health, Peer education, Awareness campaigns, Health promotion

## Abstract

In recent years, universities have demonstrated heightened recognition of and concern for students’ mental health by expanding mental health services as well as campus-wide mental health promotion campaigns. This article examines peer education –– a growing initiative on American campuses in which students work with their institution to create and deliver health education and awareness campaigns to their peers. I present findings from an ethnographic study including 30 interviews with student peer health educators and their supervisory staff, participant observation among three university programs, and content analysis of digital and print media created by peer educators. Using the framework of boundary objects (Star & Griesemer, 1989), I show how mental health education–and the very construct of “student mental health”– is produced in mental health promotion campaigns. As they co-construct mental health promotion initiatives, peer educators and their supervisors rely upon ambiguous working definitions of mental health that homogenize mental health challenges while individualizing their remedy through broad appeals to stress management. In this way, “student mental health” becomes synonymous with student success and retention, obscuring the shared and structural bases of student distress.

## Introduction

“In extending the reach of authorized mental health categories to include both clear-cut disease and vaguer, though no less serious, problems of everyday life as well as non-medical catastrophes, the term mental health became an unwieldy, even an unbelievable, odd lot–now in DSM-IV (and soon DSM-V) with hundreds of subcategories. It seems to simultaneously trivialize the most serious of medical conditions and to medicalize social problems. *I predict that by 50 years from now this category will have been abandoned.*”-Arthur Kleinman (2012:181, emphasis added)

“I think just opening up conversations and trying to make people feel as comfortable as they can to talk about it, so like using those buzzwords, I guess, to bring people in, but then really trying to curate discussions around it. I think that’s really important for promoting mental health, just talking about it, normalizing it.”-Claire, student peer educator at a large public university, white woman

Claire is a 22-year-old white woman studying public health and psychology at a large Midwestern university in the USA. I interviewed Claire at her university’s health promotion office, a modern, sun-filled room decorated with plants, fidget toys, and bowls of free wellness-themed stickers.^1^ Listing her many extracurricular activities, Claire described her role as a student peer educator working with the health promotion office to create educational mental health campaigns for her fellow students. Peer education programs are one of the most accessible ways for students to get involved with their university to support student mental health.^2^ Like many peer educators in my ethnographic study of college health promotion, Claire focused on promoting mental health through “talking about it” and “normalizing it” by “using those buzzwords.” Yet, as I observed their mental health promotion events and talked with peer educators, it became less and less clear what students meant by “mental health,” or what it is that is being normalized, promoted, or prevented through these campaigns. This paper investigates this ambiguity of “those buzzwords” through the lens of boundary objects: terms that are operationally vague yet conceptually shared across contexts, allowing for divergent definitions and uses while maintaining common meaning (Star & Griesemer, 1989).

Mental health events, infographics, and sentiments are seemingly commonplace on college campuses in the USA (Wilson, 2026) and elsewhere (Foulkes & Finan, 2024; Saltmarsh, 2016). While many of these programs have been dismissed as performative “well-being washing” (Jackson, et al., 2022) by students, researchers, and university faculty alike, I focus instead on how students and staff conceptualize and create these events, attending to their construction of “student mental health” as an object of intervention. My goal is not to examine the effectiveness of these programs but to trace how “effectiveness” is imagined and understood––it is not so much a question of “what works” but of what solutions are made possible through this problematization (Foucault, 1983, 1984; Lea, 2008). Engaging with scholarship that theorizes how policy language orients social and political practice (Cornwall, 2007; Lea, 2008; Urciuoli, 2008, 2009; Wright & McLeod, 2015), this paper contributes to a growing body of interdisciplinary scholarship engaged with the production, contestation, and experience of mental health in higher education (Alsopp et al., 2023; Anderson-Fye & Floersch, 2011; Armstrong et al., 2023; Cifuentes et al., 2025; Gordon, 2025a; Stanek and Mattson, 2024; Wiley, 2023; Wilson, 2026). My analysis of the term “mental health” as it is used by peer educators and their supervisors extends the concepts of boundary objects (Star & Griesemer, 1989) and “buzzwords” (Cornwall, 2007) to examine university mental health programming as complex, contested, and produced by various actors and priorities. By examining ethnographically how students and staff enact mental health promotion, this paper contributes to a broader understanding of the production of popular therapeutic discourse (Rose, 2007; Wright, 2010; Furedi, 2004; Illouz, 2008; Fassin and Rechtman, 2009; Carr, 2010; Weiner, 2011). After an overview of peer education and mental health programs in contemporary American universities, I explore how peer educators are trained and learn to promote the ambiguous object that is “student mental health” alongside their health promotion supervisors. I then examine their mental health promotion campaigns, arguing that their construction of “mental health” awareness and intervention collapses lived experience into self-management. This inscribes and rearticulates student distress through the imperative to de-stress.

### Student Mental Health

Peer education groups are part of a vast array of clinical, community, and outsourced mental health services introduced and expanded on US college campuses in recent decades defined by a “crisis in college mental health” (Abrams, 2022; Schwartz & Kay, 2009). College mental health services significantly expanded in response to the growth and diversification of student populations, the reorganization of mental health professions, and the neoliberal restructuring of university operations in the latter half of the 20th century (Kraft 2009, 2011; Prescott, 2008; Reinhold, 1992; Stearns, 2023). While managing student health has long characterized the social role of the American university “in fostering mature minds and healthy personalities among the nation’s youth” (Prescott, 2007:8, see also Crook, 2020; Cohen, 1983), universities have rapidly implemented novel interventions in recent years that expand access to care and distribute the responsibility of well-being across campus (Kotouza et al., 2021). This is especially the case as they seek to address public perceptions of institutional inaction, as publicized through numerous lawsuits, fluctuating college rankings, and provocative campus blogs (Napp and Nahata, n.d.; Ladany, 2022; Moss, 2022).

The expansion of university mental health programs can be understood alongside changing disciplinary policies on American college campuses. The contemporary landscape is marked by both the paternal tradition of *in loco parentis* (“in place of parents”, epitomized in campus police forces) and what Farzad-Phillips (2025) terms *in loco maternis* – “in place of mother.” *In loco maternis* encourages “student success via nurturing guidance rather than surveilled punishment” (2). Since 1970s, the relationship between students and their universities has been forged under tort law in the courts, such that “in loco parentis, it seems, now speaks the language of personal injury rather than institutional paternalism and disciplinary norms” (Cooper, 2017: 254).^3^

Universities, rather than functioning “in place” of parents, feature “parent-like” support in the form of increased student services, leadership programs, enrichment opportunities, and student development (Bickel & Lake, 1999; Farzad-Phillips, 2025; Lee, 2011), sparking critiques of a “therapeutic university” (Hermanowicz, 2024; see also Lukianoff and Haidt, 2019). Given mounting research that highlights gaps in help-seeking and care provision among students of color and LGBTQ+ students (Lipson, et al., 2022; Dunbar, et al., 2017; Eisenberg, et al., 2012), many interventions prioritize increasing access to care, thus justifying and sustaining an array of outsourced and frequently technological services such as telehealth, self-help, text-support, and self-management techniques (Gill & Donaghue, 2016; Rosenbaum & Webb, 2024; Kotouza, et al., 2021). With the availability of so many opportunities, “[i]t is now an individual’s responsibility to achieve their own quality education… shaping individuals to be citizens who are good consumers and can enter and successfully compete in the market-driven world” (Nishida, 2016:147). In an increasingly competitive and uncertain job market, students are expected to self-optimize by taking advantage of services and having leadership roles in campus clubs and organizations. It is in this context that peer education represents a leadership opportunity, a resume line, and a professional experience.

Levinson and McKinney (2013) highlight the resonances between psy-culture and the corporate university, such that North American institutions function as “psy campuses” where robust student services, mental health care, and health promotion have increasingly become regulatory marketing strategies to an ever more competitive applicant pool (see also Gottschall & Saltmarsh, 2017). Indeed, campus mental health services are not only of concern to current students but increasingly to the imagined future applicant: various college ranking publications, such as *The Princeton Review*, now feature national rankings such as “Mental Health Honor Roll” and “Best Student Support and Counseling Services” (*The Princeton Review*, n.d.). Many of today’s US college campuses now offer a wellness “suite” equipped with counselors, psychiatrists, telehealth options, crisis support, health promotion coordinators, mindfulness subscriptions, and wellness rooms, contributing to the perception of an “amenities arms race” on US campuses (McClure, 2019).

As researchers and clinicians alike find that solely expanding access to psychiatric care is neither financially feasible nor an adequate response, many campus strategies emphasize widespread health education aimed at educating students and changing campus culture. University health promotion departments are increasingly taking up mental health education, suicide prevention, and campus climate campaigns to increase students’ self-care behaviors and empower them to seek services when needed, 4 thus relying on strategies of risk reduction and self-management that increasingly define university labor (Kotouza et al., 2021; Macfarlane, 2011; Slaughter and Rhoades, 2009; See also Cottom, 2017).

### Shifting Concepts of Mental Health and Distress

A growing body of research investigating the rise in reported mental health conditions among young people has suggested a “broadening” and “loosening” of concepts of mental health among youth as well as in popular discourse about youth mental health (Bantjes et al., 2023; Glass, 2024; Jackson & Haslam, 2022; Walden & Cowen, 2024). Even as increased conversation about mental health may reflect a reduction of stigma, it may also indicate a broadening of diagnostic language, or what Haslam (2016) refers to as “concept creep” ––the expansion of psychiatric language to encompass quantitatively and qualitatively more benign experiences. Indeed, some have theorized a looping effect (Hacking, 1999) by which student dialogues, university messaging, and broader media have interacted to reshape experiences of mental distress and the terms put forth to describe them (Kvist Lindholm and Wickstrom, 2020; Chevalier, 2024. See also Foulkes & Andrews, 2023; Raikhel, 2026). Students employ dialogues of mental health and illness to navigate a myriad of social and bureaucratic contexts. This includes the use of diagnostic language and mental health literacy to navigate campus services such as counseling or disability services (Cifuentes et al., 2025) and to demonstrate challenges in appropriate dialogue that is “bureaucratically legible” to the university (Crook, 2020). Other research has considered how students use mental health terms as a form of social utility: to distance oneself from privilege (Stanek and Mattson, 2024), obtain social capital for certain valorized conditions (Armstrong et al., 2023), affirm one’s identity and shared experiences (Wiley, 2023), and seek a sense of belonging through performing academic rigor (Wilson, 2026).

The term “mental health” has long been and continues to be a contested and ambiguous concept (Rosenberg, 2007), simultaneously denoting a state, a dimension of health, a social movement, and a professional object (Bertelote, 2008). Just as Claire, in the opening vignette, endorsed the importance of normalizing and talking about “it”, imperatives to promote, prevent, and raise awareness seldom articulate the assumptions embedded in the term mental health, in what ways it can be intervened upon, and to what ends (Fey & Mills, 2021). Moreover, public discourse on mental health fails to distinguish “good” mental health from “the rather different category” of mental illness or disorder (Malla & Gold, 2024:126).

From its inception, the concept of “mental health” has been necessarily ambiguous. As Doron (2015) writes of the origins of psychopolitics,

“it should be emphasized that the notion of “mental health” is primarily characterized by the way in which it unites different activities…This was part of the concept’s function from the outset: in order to establish a field (the “mental health field”) that could bring together actors with fairly heterogeneous roles, the meaning of the category had to remain largely ‘underdetermined’” (2015:5).

In this way, the concept functions as a boundary object (Star & Griesemer, 1989). Boundary objects are “both plastic enough to adapt to local needs and the constraints of the several parties employing them, yet robust enough to maintain a common identity across sites” (1989:393). Boundary objects inhabit multiple social and professional worlds, structuring relations between them despite differential uptakes of their definitions. For example, different campus offices are able to draw upon various conceptions of mental health while retaining a shared conceptual basis–to the marketing office, “mental health” is enacted as smiling faces and a vibrant campus community; to the dean of students office, “mental health” might be enacted as end of year dropout rates and leave of absence statistics; for the counseling center, “mental health” might be enacted as appointment wait times or referrals to outside providers; for dorm resident advisors, “mental health” might be enacted as a self-care bulletin board; for the instructor professor, “mental health” might be enacted in questions about whether to report a student. As a “buzzword” (Cornwall, 2007), mental health affectively connotes equality and inclusion without meaningfully acknowledging complicity with structural inequity in the university (Ahmed, 2012). Buzzwords, Cornwall argues, “gain their purchase and power through their vague and euphemistic qualities, their capacity to embrace a multitude of possible meanings, and their normative resonance” (2007:472). Like keywords (Williams, 2011[1976]), they are “warmly persuasive” and invite no disagreement (Cornwall, 2007:472; see also Syvertsen, 2025). While campus offices may operationalize mental health variously, the importance and the urgency of mental health is maintained and sustains relationships between campus actors. Though, as Doron (2015) writes, such an undetermined definition stifles deeper engagement, making mental health “a ‘norm of action’ imposed from the outside, generating resistance rather than support” (2015:5; See also Ahmed, 2012). Importantly, as boundary objects facilitate certain systems and solutions, others are rendered invisible, unnecessary, or unthinkable. As I explore how the term mental health is used, I attend specifically to how it maintains a broad imperative of student self-management and forecloses deeper engagement with the structural components of student distress and the unique individuals that a “crisis in college mental health” represents (Aftab & Druss, 2023).

### Peer Health Educators

Peer-based mental health support has been taken up variously in university contexts, often as peer education, peer counseling, or peer support groups (Pointon-Haas, et al., 2023, Humphrey, et al., 2022). In psychiatric and community care contexts, “peer” refers to a person with lived experience of mental illness and/or service usage who draws from their experiential expertise to provide support for another (Dixon et al., 1994; Myers, 2015). While the term “peer” and the function of “peer support workers” remain complex issues (Dixon et al., 1994; Penney, 2018; Voronka, 2017), the term “peer”, in most cases, recognizes lived experience of mental health challenges.

Frawley and colleagues (2024) describe peer support in UK universities as the mobilization of “professional exes” (Brown, 1991) by which a student is transformed “from a liability to be supervised to an asset to be utilized” (Frawley, et al., 2024:363). Through peer support programs, students who have experienced mental health issues are embraced as policy and self-help entrepreneurs whose lived experience and expertise coalesces well with professionalized narratives of mental health intervention (Frawley, et al., 2024:363). In other words, narratives of recovery are instrumentalized for campus risk reduction and narratives of illness replaced with well-being (Aubretch, 2012). When students must constantly self-optimize for future stability (Demerath, 2009), the role of “self-help entrepreneur” presents an opportunity to leverage care and community for marketable qualities.

While most peer support programs (Byrom, 2018) explicitly name students’ lived experience as a mode of intervention and a source of support, the peer education programs I studied in the United States instrumentalized student experience much more narrowly; “peer” was defined as “a person of equal standing with you in a group,” and a peer educator as “a trained student in a helping position who encourages healthy choices.” In contrast to the use of “peer” in the UK university and many psychiatric care contexts, “peer” functions among my interlocutors as “not staff.” Their college experience, not their mental health histories, are instrumentalized for health promotion: Peer educators are the “foot soldiers” and “brand ambassadors” that bring the student wellness office to the student body, as one supervisor framed them. Peer education programs thus offer a rich opportunity to explore how mental health promotion functions with a rather different utilization of “peer” identity and utility.

Peer educators are responsible for creating and executing health education and promotion initiatives in support of broader institutional goals, namely increased utilization of an array of campus health services and self-care behaviors.^5^ While the staff members I interviewed recalled fragmented histories, literature places the origins of peer education on college campuses throughout 1960s–70s counterculture movements as students shared alcohol education, reproductive health care, and mental health support (Humphrey, et al., 2022). As they were co-opted into institutional programs alongside the expansion of student services in 1980s and 1990s, peer education programs transitioned “from self-educated students responding to campus health issues to state-of-the-art health education and motivational models” (Sloane & Zimmer, 1993:241; cf Turner & Shepherd, 1999). While typically inclusive of many facets of health or “dimensions of wellness,” peer educators have become increasingly focused on mental health programming, including self-care, stress management, and self-assessment (Lemon & Wawrzynski, 2021); peer educators who specifically focused on mental health are the focus of this study.

In my fieldwork and in broader literature (Cofer et al., 2022; Sloane & Zimmer, 1993), peer education programs are heralded for their transformative effects on both the student leaders facilitating these events and the student body receiving them, so much so that many of their staff supervisors had been peer educators themselves and pursued public health because of this experience. Not only are they financially preferable to psychiatric services, but campus-wide intervention programs tap into existing wells of student labor and demonstrate to the student body that their voices are represented. Marketed as “high-impact experiences,” students gain leadership opportunities, oral presentation skills, and resume boosters. Fewer than half the programs surveyed in this study financially compensated their peer educator. It is not uncommon for these students to assume responsibilities that would otherwise be performed by salaried health professionals, indicative of a broader decline in higher education funding leading universities to balance budget cuts with the growing desire for student leadership opportunities (Farzad-Phillips, 2025). The students I interviewed, more than half of whom were studying in health-related fields, understood peer education as an important step toward a career in health professions. Many joined the group out of a desire to raise awareness of mental health on campus given the apparent state of inadequate campus mental health services, especially, students noted, in the wake of the COVID-19 pandemic.

### Methodology

Student peer educators are trained by and work closely with their campus health promotion office, making peer educator training and practice a critical site of reciprocal knowledge: where students are taught institutional understandings of health and wellness and where students’ needs are represented to health promotion staff. Methodologically, this project builds upon ethnographies *of* rather than merely *in* universities (Anderson, 2023), situating universities not as the mere context in which student experience takes place but as active participants in the social, economic, and political forces that structure students’ daily lives (Pötschulat et al., 2021; Shumar & Mir, 2011). Institutional ethnography (Smith, 2005) and meeting ethnography (Sandler & Thedvall, 2017; Schwartzman, 1989) guided my approach to examine how institutional values are translated and contested across student-staff interactions.

To better understand how staff and students constructed their interventions, this ethnography consisted of (1) 17 interviews with university staff and thirteen with undergraduate students; (2) participant observation at three universities and one peer education conference; and (3) media analysis of university websites, peer education training materials, and the pamphlets, stickers, and stress balls produced by these programs.

Interview participants were overwhelmingly white women, demographic factors that have historically defined the field of health promotion. Twenty-three participants identified as women, four as non-binary or gender non-conforming, and three as men. Participants’ self-identified racial and ethnic identities include: 21 as White, five as Asian or Asian American, three as Black or African American, and two as Hispanic/Latinx and White.

Fourteen universities are represented across the interviews in this study. To recruit, I identified and contacted peer education programs at more than 50 colleges across the United States that varied in institution size, type, and location. After some initial responses, I continued to recruit in areas underrepresented in my sample. Staff at fourteen institutions consented to participate in the study and forwarded the study information to their current peer educators. Of these fourteen institutions, seven were public and seven were private; eight were research intensive institutions^6^ and three were liberal arts colleges. Geographically, they represent: four Southeastern, five Midwestern, three Northeastern, one Southwestern, and one Western US universities.

Participant observation occurred from May 2024 to January 2025 in the health promotion offices of three universities in the Midwestern United States.^7^ These sites were chosen to reflect diversity in institution type, size, and geographic location. University 1 is private, suburban, with approximately 8,000 undergraduate students, and its peer education group has four students who focus on all areas of wellness; University 2 is private, urban, with approximately 12,000 undergraduates, and has a peer education group of 30 students, six of whom specialize in mental health; University 3 is public, rural, with approximately 18,000 undergraduates, and has a peer education group of twenty students. Institutional site permission was collected in addition to student and staff informed consent. At each university, participant observation took place during health promotion staff meetings, student training sessions, peer educator weekly meetings, and the programs they hosted. Importantly, my observations centered the interactions between students and staff as they created and debated social media posts, raffle prizes, and mindfulness activities; this is an ethnography of peer educators just as much as a study of the institutional offices they are embedded within.

My personal and professional identity facilitated a proximity, or an “insider” position, which directed my access and perspective (Hodkinson, 2005). My relationship to my interlocutors was informed by my proximity to their identities and experiences: I am a young white woman and former peer educator roughly the age between peer educators and their supervisory staff, easily (and often) mistaken at campus events as belonging to one of the two participant groups.^8^ Staff, many of whom had been peer educators themselves and pursued public health because of it, recognized my familiarity with the procedures of peer education; I was often invited to review training materials, attend staff meetings, and give feedback on events. In a number of my interactions with both students and staff, my interlocutors referred to me as the “expert” on mental health because I had received more training about mental health in a medical anthropology program than they had in their public health or peer education training.

#### Findings

### Peer Education as Public Health Modality: Training

All programs I encountered required training before students could become peer educators, ranging from 6 h to 40 h or even an entire semester.^9^ This training, which in my observations was created and delivered by the health promotion coordinators who supervised the groups, focused on equipping students with the tools of behavioral change and the skills to effectively convey health information. The roles of peer educators, as described during one training I attended, include to “correct misperceptions of behaviors/choices, promote healthy choices with facts, and model healthy behaviors.” Much of this work is positioned as inciting behavioral change––persuading students to get more sleep, eat more nutritious meals, practice meditation, study smarter, drink more water, and assess when they need to seek help (Bunton et al., 1995; Lupton, 1995) Peer educators were taught public health theories such as the Transtheoretical Model and the Health Belief Model, among others. For example, peer educators were presented with a hypothetical student who may be in the “precontemplation” stage of the Transtheoretical Model––someone who is not yet aware of the behaviors they ought to change, like procrastination, high caffeine intake, or poor sleep hygiene. It is critical, reminded their supervisors, not to approach someone who is in “precontemplation” with information that would better suit someone in “action stage.” In this way, peer educators were taught to see their roles as fashioning information to inspire behavioral change: A significant part of their training was reserved for sharpening students’ skills to *deliver* health education, practicing, for example, using the correct institutional brand font and colors, creating flyers, and proper social media posting etiquette.

While their staff supervisors insisted on developing “data-driven,” educational interventions, peer educators were often preoccupied with designing an engaging and fun event, given that the student body attends these programs voluntarily. 10 Indeed, the vast majority of my observations involved watching peer educators debate event flyers, sticker designs, raffle prizes, and event names. Supervisory staff expressed frustration with this practice (“this isn’t how public health work is done!”) and lamented that it typically resulted in stress relief events– “the low hanging fruit of health promotion” –because these events appeal to the largest number of students. However, peer educators’ preoccupation with the aesthetics of health information makes sense given their limited means of measuring effectiveness; evidence of a “successful” program often only comes in the form of high attendance, the number of items distributed, and the self-reporting of how well attendees enjoyed the activity. And a “successful” event was critical, since supervisory staff used this data to advocate for institutional resources. Thus, “student engagement” becomes synonymous with “student well-being” (Callard, 2025), both in how it is measured and how it comes to be imagined. The difficulty demonstrating effectiveness was made clear in Shannon’s, a health promotion coordinator, reflection: “it’s hard to show the impact of the progress that we’re making. I can’t go to the provost and be like, ‘I just stopped all these potential suicides’....”

Notably, since many of these peer education programs focused on various areas of health with topic-specific subcommittees, they were often trained as a group on all areas of health. What differentiates mental health from other topics, though, is its relative lack of epistemological frame or template. While nutrition committees learned of caloric intake, food deserts, and body neutrality; gender-based violence groups learned best practices of consent, bystander intervention, and Title IX procedures; and alcohol and other drug committees were trained in state law, drug safety, and alcohol content in drinks, mental health groups were given little to no framing to approach education or behavioral change. Some programs provided opportunities for peer educators to learn more about mental health interventions by attending campus crisis training or reading up on suicide prevention practices such as QPR (Question, Persuade, Refer), but these were often optional and not expected to be part of the peer education programming. The meaning of mental health, and its ability to be intervened upon through public health, was taken to be self-evident to such a degree that only one training I encountered featured any substantial information on mental health, and this was the World Health Organization’s official definition and a presentation of prevalence statistics. Across my fieldwork, information about mental health was often presented in the form of staggering statistics from national samples or their own institution’s “Healthy Minds Survey” ––a survey conducted annually across hundreds of campuses to assess students’ self-reported experiences. Such surveys are often the source of infographics warning that upward of 80% of students experience anxiety, stress, and depression, carrying the credibility of diagnosis but not the validity of clinical measures (Glass, 2024:789–90).

Even as they learned about educational and behavioral change models, knowledge of mental health––of issues, etiology, treatment, or experience––did not emerge as a necessary component to health education. Rather, the tools for promoting health could be—and were—distinguished from knowledge about health. Sarah, a white woman and student leader of her group, likened this to her public health major, reflecting:

“We talk about health in each class, like, it’s definitely brought up a bunch, I think. But we're not, like, focusing just on that, and we don’t have units of mental health. I think so much of public health right now, for me, is just like, how to promote. It’s not even putting into action, it's just kind of talking about the logistics of it and the systematics of it.” (Student leader of peer education group at a midsize private university, white woman)

Moreover, while they discussed at length the means of delivering mental health education and measuring its impact through engagement, the content of such information and the goals of their programs were largely taken for granted. Staff members, most of whom had degrees in public health, affirmed their belief in health promotion tools. Rachel, a Black woman and health promotion coordinator at an elite private institution, put it this way:

“We need to take a public health approach. Because we know that public health things work. Like CDC [Centers for Disease Control]. We can eradicate polio. How can we, you know, take that formula and apply it to mental health? We’re still figuring out if this is moving the needle enough.” (Health Promotion Coordinator at a midsize private university, Black woman)

A few months later, I interviewed Rachel again after observing her peer education group over the academic term. I’d been growing frustrated with how little I had been able to talk with students or staff about how they actually understood mental health; my conversations with staff had been dominated by their most effective giveaways (speakers and plushies), their best strategies to manage peer educators (compensation, attendance policies, accountability measures such as regular updates), and their most popular campus events (de-stressing carnivals, therapy dogs). When I explained this to Rachel, she thought for a few moments and admitted: “I’m not really sure you can promote mental health through behavioral change. What is mental health? What are the behaviors?” In this instance, Rachel laid bare the frail epistemological frame of mental health intervention, posing the question I had been asking peer educators: *what is mental health?*

### Broadening the Scope: Talking About (Talking About) Mental Health

“Let’s plan our de-stressing event!” says Sarah as she begins the weekly peer education meeting at her private Midwestern university. The first few times I heard peer educators talk about these kinds of programs, I failed to register the long “e,” leaving my notebook full of question marks that peer educators wanted to “promote distress.” Even as I attuned to this double inflection of “distress” and “de-stress,” it was often difficult to identify the valence with which students and staff talked about mental health in their conversations and programs. Listening to peer educators brainstorm their initiatives or sitting on their side of a tabling event handing out pamphlets, I noticed how, in the same breath, peer educators endorsed both preventing and promoting mental health. “Mental health” implies both a capacity and a detriment, such that the term captures, but does not make explicit, both well-being and illness (Frawley, et al., 2024). Rhetorically, mental health functions as a container (Glass, 2024) or an “umbrella category” (Jackson & Haslam, 2022), describing affects to be both achieved and avoided. Similar unspecified “buzzwords,” as Claire put it, could be found on recruitment flyers that call out to applicants who are “passionate about gender-based violence and alcohol” or at tabling events where peer educators accidentally expressed their “passion about suicide.” Such slippages between “distress” and “de-stress” or “suicide” and “suicide prevention” indicate the vagueness and indeterminacy of these efforts.

Ella, a Black woman and pre-med student at a large, rural university, similarly emphasized the importance of creating spaces to speak openly, expressing how “talking about mental health is a way to educate about mental health.” Characteristic of contemporary psychological personhood that encourages verbal self-reflexivity and self-surveillance (Adams et al., 2009; Furedi, 2004; Lupton, 1995; Weiner, 2011; Weinberg, 2021), the imperative to “talk about” mental health underpins the majority of contemporary health promotion, stigma reduction, and mental health literacy campaigns. Though, as is certainly the case here, these campaigns often rely on ambiguous terms of mental health and are restricted to mental health issues that are “more recognizable, safe, and knowable than others” (Frazer-Caroll, 2023:40). When I asked peer educators to explain what they meant by mental health or how they understood their work to address it, nearly all expressed confusion, surprise, and at times discomfort at the very question. I interviewed Sarah, a white woman and the student leader of her group, at her student wellness center after a few months of attending her group’s meetings and events. She described mental health in this way:

“It’s hard, because I am a public health major. Oh, gosh, I should actually know. It’s hard … well-being? Day to day function? This was a dreaded question. Like the state? I don’t know. Because, like, what is healthy?”

While her questioning of what is healthy could be read as a critique of pathologization and the relativity of health, Sarah’s and others’ consistent inability (and the apparent dread experienced when trying) to operationalize how their programs addressed mental health reflect a broader ambivalence and uncertainty with which health promotion campaigns approach student mental health. It also reveals how this question becomes unnecessary and out of place; it seemed that students and staff working in college health promotion saw “mental health” as a vast and ubiquitous issue which required no need for specificity or meta-analysis (in later sections, I explore the rhetorical utility of this ubiquity). In this way, the term functions for peer educators as a boundary object––allowing the term “to be used flexibly as a justification for myriad purposes—an a priori good endowing such interventions with ethical intent” (Frawley, et al., 2024:364). Thus, “shared representation might be quite vague but at the same time quite useful” (Star, 2010:607). Even as they struggled to and did not see the purpose of defining it, peer educators’ endorsement of “talking about” and “raising awareness” of mental health is indicative of a broader ethical project––*clearly* student mental health is an issue and *clearly* we ought to do something about it. In college health practice, the term mental health can be deployed to evoke a sense of crisis, diagnostic severity, and specialization while maintaining an appeal to the everyday life stressor and the everyday college student. As Frawley and colleagues (2024) write, “loosely defining problems can be rhetorically advantageous, as a too-restrictive definition risks downplaying the problem’s size and thus necessity for action” (355).

This impulse to broaden the scope of mental health to appeal to more students is reinforced through exclusionary recruitment processes that discourage and exclude students with lived experience as well as through the gendered nature of health promotion practice: The majority of peer educators are white women, despite efforts for more diverse recruitment. As mentioned, the use of the word “peer” in university peer education denotes a shared student status in contrast to staff, who are more typically the messengers of health information. When asked how students’ own mental health informed their work as peer educators, staff supervisors quickly dismissed the possibility of students using their mental health experience as a means of relationality. In my interviews with staff, a peer educator’s own mental health emerged only as something that impedes their ability to perform their roles. Staff had not considered having students share or draw explicitly upon their own experiences with mental health issues; it was their experiences related to being a college student that forged their relational utility. Interestingly, a number of supervisors I interviewed considered it to be exploitative for peer educators to share about their own mental health. For example, Rachel, a Black woman and health promotion coordinator at an elite private institution, contrasted peer education with identity-based work, saying:

“It’s exploiting. It can be, it comes across as exploiting to me, at least, like exploiting somebody being like, ‘mmm, so you experience depression and have ADHD? We need you to go out and talk to students who have the same experience.” It’s kind of like, whoa, huh? Whoa? That’s odd. Why would you pick me out like that and just use my trauma for quote unquote ‘good? ’.. Who’s to say that’s even helpful? Find an expert instead.”

Rachel was not alone in her assertation that peer support was not identity-based or that experience with a mental health condition did not offer expertise. Other staff I spoke to interpreted disclosure as selfish and self-serving, or even unintended and accidental, like a slip of the tongue that students would later regret sharing. Overall, disclosure of one’s own mental health was broadly considered inappropriate.^11^ Often, students who spoke about their own mental health in interviews were not selected; at one school, students who currently receive counseling services at the institution are barred from applying at all. The students who apply and become peer educators engage in the reproduction of ideas of wellness that emphasize consumption (stickers, bath bombs, journals, and coloring books), and self-management (sleep hygiene, mindfulness, self-assessment, and biohacking) (Sobo, 2024). Moreover, the visions of self-care offered through the university and curated by peer educators continue to individualize wellness, minimizing possibilities for rest, resistance, and community care (Gordon, 2025a; Vázquez, 2025).

While a full analysis of the meaning of “peer” is beyond the scope of this article, it is important to emphasize that when mental health is discursively broadened to encompass more experiences, it becomes more difficult, even unnecessary, to acknowledge the specific individuals and experiences captured in the term “mental health. “It becomes possible to promote mental health without ever explicitly talking about one’s experiences. The next section analyzes how peer educators keep the scope broad in order to put mental health promotion into practice.

### Marketing Mental Health

Many health education campaigns began with a broad reference to the statistical severity of mental health conditions on campus, justifying to their supervisors and making the scale of the problem visible to participants. At the same time, their interventions attend to mild forms of emotional distress. Even as they cite statistics that the majority of students meet the threshold for psychiatric diagnosis or that suicide is the second leading cause of death for college students, their conversations, program activities, and interventions rest on the imperative that they appeal to the broadest audience–and the apparent assumption that their audience does not embody those same statistics. For example, when I asked a student why they did not do events on more specific topics, like medication management or obsessive-compulsive disorder (OCD), they told me that not enough students need this information or would attend an event focused on it. Thus, while they might cite diagnostic statistics, their initiatives rarely used diagnostic language or directly named mental disorders.

“Our most successful stuff has been focusing on a more generalizable stress management angle, given that all students are stressed,” said Wren, a white, gender non-conforming peer educator at a prestigious private university. Wren joined their school’s peer education group thinking, they would discuss more serious topics like self-harm or suicide and contribute to shaping campus policy. While admitting that they did not consider their programs to be a major form of intervention, Wren nonetheless defended de-stressing events because “this might be the only ten minutes these students spend taking care of themselves today.” Wren was not alone in describing a desperate sense of convincing students to do something to take care of themselves. As a health promotion coordinator at an elite university put it:

“A lot of our work ends up taking the form of trying to convince students that health is something to desire. Especially at this institution, we find it really hard to get some of these messages to them unless you appeal to them as students. It’s not like, ‘hey, if you just get more sleep, you’ll feel better, right?’ It’s very evidence-based, because we’re training scientists and scholars.” (White woman, Staff supervisor at a mid-sized private university)

This supervisor adds to Wren’s sentiment that the work of health promotion is largely to *convince* students to take up certain behaviors and care about their health. Just as Wren found stress management to be the most effective “angle,” the most ubiquitous strategy used by the groups I observed was to link self-care with academic success. When they are “training scientists and scholars,” convincing students requires appeals to their academic success and as a form of knowledge-building.

Keeping the meaning of mental health broad allows for it to encompass a wide range of behaviors, experiences, and problems as amenable through mental health intervention. Addressing mental health as and through appeals to academic stress both ensures the greatest number of attendees and keeps the meaning of “mental health” ambiguous enough to be relevant to each of them. But, when mental health is only or mostly articulated as an academic issue, academic struggles are inevitably situated as preventable through “better mental health practices” (Aubretch, 2012).

**Fig. 1:**
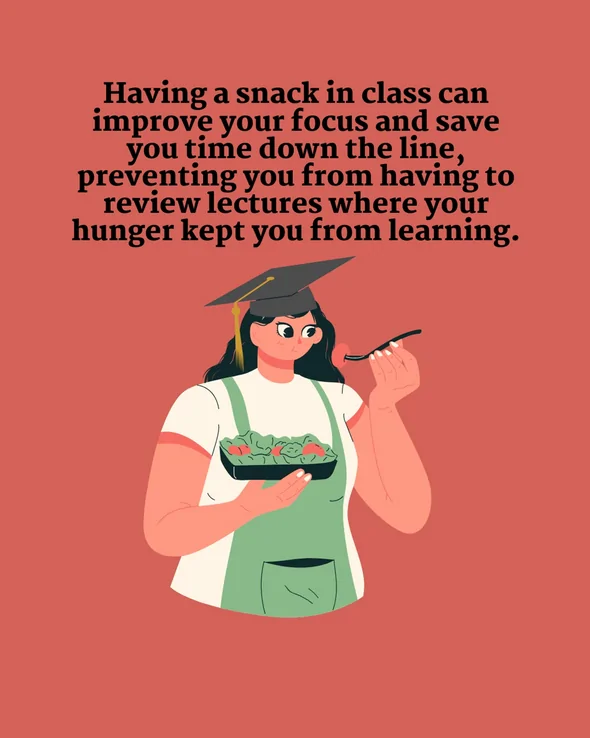
A recreated Instagram post made by peer educators. The background was chosen by searching “eating disorder awareness” on Canva, the graphic design website used by nearly all peer education groups I encountered

For example, one peer education group had created a social media campaign for eating disorder awareness week, in which they situated healthier nutrition and eating practices in association with academic outcomes. The group made an Instagram post (Figure 1) that suggested that “having a snack in class can improve your focus and save you time down the line, preventing you from having to review lectures where your hunger kept you from learning.” Other parts of the campaign warned against the harmful effects of restrictive eating on learning and memory, which could hinder academic performance.

**Fig. 2:**
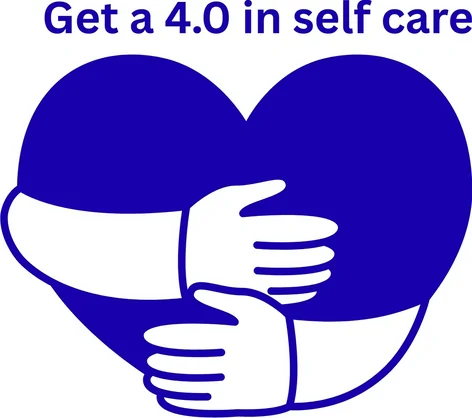
Recreation of a sticker found on a peer educators’ notebook that reads “Get a 4.0 in self-care”

Other materials encouraged students to get enough sleep in order to perform well on their examinations. A sticker (recreated in Figure 2) I found on a student’s notebook, from one of the most widely used telehealth services by universities in the USA, reads: “Get a 4.0 in self-care,” likening self-care to academic success, as though self-care should be measured and achieved. Others read “turn your stress into success,” exemplifying how mental health is animated as itself an academic tool.

Failure to take care of oneself (i.e., not eating enough) directly interferes with one’s academic success (i.e., hunger keeps you from learning). When academic failure is framed as preventable through better mental health, this inherently frames academic failure as a mental health issue, and mental health issues as matters of academic concern. Importantly, it also obfuscates the connection between academic or institutional practices and student well-being in the first place. An individual’s capacity for self-improvement supersedes the context of economic insecurity, institutional austerity, an uncertain job market, and political instability. Moreover, “university ideas of wellness continue to socialize students to think of mental health as a matter of individual, personal responsibility disconnected from social structural forces” (Weinberg, 2021:7), thus articulating mental health as something that both can and should be acted upon through the individual body and mind (Aubretch, 2012; Blackmore, 2009; Dolmage, 2017).

### Discussion: Promoting Mental Health in the Contemporary University

As it encompasses a wide range of affects and experiences, the term mental health allows peer educators to draw upon the ethical urgency of a widespread mental health crisis that justifies and sustains interventions, while at the same time enlisting all students as in need of mental health intervention: “If everyone has mental health, then everyone has mental ill-health too” (Frawley, et al., 2024:365). Mental health is evoked as both a capacity and a deficit of well-being, such that prevention of ill-health and the enhancement of well-being emerge as inseparable aspirations and simultaneous endeavors; as many peer educators uttered, it’s to be both promoted and prevented. To speak of health and normality is always to position them in relation with their imagined corollaries. As Canguilhem (2008) writes: “we can’t say that the concept of the ‘pathological’ is the logical contradictory of the concept ‘normal,’ for life in the pathological state is not the absence of norms but the presence of other norms” (131). It may be that peer educators’ use of “mental health” remains so ambiguous because it contains the normal and the pathological, the healthy and unhealthy—a paradox that mirrors their imagining of a homogenous student population in crisis yet in need of individual self-management and self-assessment.

By promoting mental health through appeals to academic success, mental health is affirmed as a constant ethical, psychological, and, increasingly, an academic project. As suggested by Rosenbaum and Liebert (2015), the term “mental health” functions as both a baseline and a goal among students—marking any deviation from the norm in need of intervention (190). Distress is homogenized as an unavoidable side effect of productive academic life, and, in the process, it is naturalized as a precondition; thus, the best forms of mental health care are reactive, seeking to shape the psychological interior of students in ways that work within the system, rather than inspiring critique of the systems in which students’ mental distress emerges (Metzl & Kirkland, 2010).

It is also worth considering how this broad discourse of mental health stands in for more explicit discussion of lived experience. Itself an ironic idiom of distress, the term serves as a broad cultural category and an accessible means to express the significant forms of doubt, discomfort, loneliness, and certainly anxiety and depression, that many college students experience. Paradoxically, the language of mental health “offer[s] an opportunity to be vulnerable without being vulnerable” (Armstrong, et al., 2023:991), signifying, but not making known, that students are struggling. When the term is used ambivalently and ambiguously, students and university staff alike become less equipped to talk about student experiences in explicit terms and to discuss the structural foundations of this distress, articulating what are likely shared experiences (overwhelm, loneliness, and fear of failure) as distinctly individual phenomena (Walden & Cowen, 2024:805; see also Wilson, 2026). Even amidst widespread invitations to speak openly about mental health, “the collective conclusion being reached seems to be less of solidarity and more of epidemic: everyone has mental health problems, and they all need help that is not available” (Glass, 2024:791).

The co-option of peer support into higher education and its institutions has long been underway. The ethical imperative underpinning mental health awareness and promotion continuously justifies the broadening of mental health interventions into more and more domains of student experience, and student experience into academic affairs (Farzad-Phillips, 2025). It also sustains the rapidly growing outsourced and technological interventions introduced on college campuses in the name of increasing access to care (Rosenbaum & Webb, 2024). The ambiguous use of mental health as a boundary object aids in this process, marking more experiences as open to intervention and positioning new markets of health services as both irresistible and inevitable (Frawley et al., 2024). Moreover, when student mental health is framed as primarily an issue of access to services, the individual procurement of psychological services triumphs over careful consideration of the context in which those experiences arise (Rosenbaum & Webb, 2024).

## Conclusion

The term mental health continues to fill the headlines of university blogs, research articles, and national news outlets as rising numbers of college students report significant levels of stress, anxiety, depression, and loneliness. Its ubiquity has directed attention to what are objectively widespread forms of student distress. It has expanded resources, occupied boardroom agendas, and formed campus offices of student flourishing and funded deans of well-being. This urgency has rapidly expanded institutional capacity for health promotion and driven thousands of students across the country to become peer educators on their campuses. Increased awareness is undoubtedly a positive shift, but it demands interrogation of the experiences, solutions, and subjectivities this awareness makes visible and those it obscures. As it seeks to mean everything, though, the term “mental health” functions to mean nothing at all, stifling consideration of the experiences of student distress captured in ethical imperatives of “student mental health” (Glass, 2024; Rosenbaum & Liebert, 2015). As a result, mental health interventions depend upon broad conceptualizations of stress and mental health that constrain their ability to act meaningfully upon the shared and structural bases of student distress.

Rather than dismissing mental health interventions or their organizers as performative, this research suggests a need to interrogate how discourses of stress and well-being are produced through campus collaborations. Attending to how mental health is constructed as an object of intervention helps elucidate the constraints in which well-intentioned students and staff go about creating community programming–how what began as grassroots lived experience peer model has been transformed into an institutional practice that alienates “student mental health” from the students it purports to care for. As the term continues to change and if it, as Kleinman predicts, is eventually abandoned, we must closely attend to how discourses of mental health are created, sustained, and transformed.

## Data Availability

No datasets were generated or analyzed during the current study.

## Footnotes

1 Many American universities have an “Office of Health Promotion” or feature health promotion services in their counseling or wellness centers. Usually, they employ a small number of staff with graduate degrees in public health and student development. They are primarily responsible for collecting data about student health, marketing health services and events to the student body, and building relationships between students and the health center. In this article, “health promotion” is used to refer to university-affiliated programs related to promoting well-being through education, empowerment, and community activities.

2 Many informal and/or student-run forms of mental health awareness, support, and mutual aid continue to exist and form on college campuses. Peer education programs specifically work in tandem with university health promotion operations, making these programs sites of student-institution partnerships.

3 For a more comprehensive history of student-university relationships, see Bickel and Lake, 1999; Lee, 2011; Hogan and Schwartz, 1987; Suriel et al., 2024.

4 These are frequently in partnership with national non-profit initiatives such Active Minds, The JED foundation, and the Green Bandana Project, which each aim to support students and staff to develop communities of care. See (Active Minds, n.d.; Jed Campus, n.d.; The Green Bandana Project, n.d.) 

5 While it is beyond the scope of this paper to examine these institutional goals in depth, I consider increased utilization and self-management to be part of a larger neoliberal shift in higher education toward risk-management and security (Doyle, 2015; Kotouza et al., 2021; Slaughter and Rhoades, 2009). Through my observations, I understood “utilization” to encompass the likelihood that students would be able to identify when they needed help, be knowledgeable about the resources available, and act upon this to seek care. For a critique of how university mental health services require students to be rational, reflexive actors, see Gordon 2025b.

6 Universities in the United States are classified by the Carnegie Classification. Eight universities in this sample are classified as “R1” institutions, indicating very high research funding and large number of doctoral students.

7 These programs differed operationally in things such as the number of peer educators, compensation structures, type of programs prioritized (workshops, tabling events, social media campaigns, peer support), and other logistical components, though their approaches to mental health promotion drew upon similar logics, rooted in shared emphases of public health and student engagement. Thus, while my analysis contains mentions of how these logics were differently manifested at particular institutions, I do not make any significant comparisons between institutions or peer education groups.

8 I prefer the language of “proximity” to insider ethnography because, as many scholars have noted, the boundaries between “insider” and “outsider” are fluid and contested. Moreover, while it facilitates relationships, access, and experience of research, the position of an “insider” or “native ethnographer” does not enable an objective, clear view of social phenomena. All research is limited by and unique to the researcher, making all perspectives partial, situated, and subjective (Haraway, 1991; Wolcott, 1994, 1999).

9 A considerable market is growing for the training and program curriculum for college peer educators. The organization NASPA, which serves as the corporate and bureaucratic home for student affairs professionals across American universities, offers “Certified Peer Educator” training featuring much of the same public health models and marketing principles making up the training I attended. Due to copyright, I was unable to attend any sessions using this training structure or to officially analyze any training materials. In my conversations with staff who had used the Certified Peer Educator (CPE) training in the past or had considered implementing it, the consensus seemed to be that the credential of a certified peer educator was the only difference between NASPA training and the in-house training developed by each school. As part of my fieldwork, I attended and presented at the NASPA Peer Education national conference.

10 While student peer educators were responsible for designing these initiatives, each step was supervised by health promotion coordinators who would assess the suitability, feasibility, and usefulness of the initiatives. Students frequently proposed programs such as plate-breaking therapy, therapy animals, arts and crafts, and other fun activities. Though, as noted here, the appeal of these programs relied on the engagement they are likely to generate, not necessarily the information they convey or the behavioral change they incite.

11 When I asked one supervisor to reflect on why this might be the case, she related it to work she had done as an advocate at a sexual violence resource center; advocates, she told me, were instructed never to disclose if they had experienced sexual assault, even if a client asked. Disclosure, in this case, was considered self-serving and to complicate the relationship between advocate and client.
